# Testicular intestinal-type adenocarcinoma without an identifiable extratesticular primary site: a case report and literature review

**DOI:** 10.3389/fonc.2026.1926619

**Published:** 2026-09-03

**Authors:** Jinrong Tang, Heng Jiang, Xiaoke Zeng

**Affiliations:** Andrology Unit, Department of Ultrasound Diagnosis, The Second Affiliated Hospital of Army Medical University, Chongqing, China

**Keywords:** immunohistochemistry, intestinal-type adenocarcinoma, magnetic resonance imaging, partial orchiectomy, somatic-type malignancy, testicular cancer, ultrasonography

## Abstract

**Background:**

Testicular intestinal-type adenocarcinoma is an exceedingly rare malignant neoplasm characterized by intestinal epithelial differentiation and accounts for only a minute proportion of testicular malignancies. Its imaging features remain insufficiently characterized and may overlap with those of other testicular tumors, particularly seminoma. When intestinal-type adenocarcinoma is identified in the testis, metastatic adenocarcinoma from the gastrointestinal tract or other extratesticular sites must be excluded before a definitive diagnosis can be established.

**Case presentation:**

We report a rare case of a 33-year-old man in whom a left testicular nodule was incidentally detected during a preconception health examination. The patient had no scrotal pain, swelling, weight loss, altered bowel habits, hematochezia, or other gastrointestinal symptoms. Serum tumor markers were all within normal limits. Imaging evaluation, including ultrasonography and MRI, revealed an irregular hypoechoic nodule measuring approximately 9.7 mm, with peripheral calcifications, prominent peripheral vascularity, and peripheral rim enhancement. Histopathological examination and immunohistochemistry confirmed intestinal-type adenocarcinoma. The patient underwent left partial orchiectomy. Upper gastrointestinal endoscopy, colonoscopy, and contrast-enhanced abdominal and pelvic CT revealed no evidence of an extratesticular primary tumor. No signs of recurrence or metastasis were observed during 6 months of postoperative follow-up.

**Conclusion:**

Testicular intestinal-type adenocarcinoma lacks specific clinical manifestations, and serum tumor markers are usually within normal ranges. On imaging, it may present as a small intratesticular nodule with peripheral enhancement and should be differentiated from seminoma, mixed germ cell tumor, sex cord-stromal tumor, and metastatic adenocarcinoma. Through a literature review, our report provides a comprehensive summary of its multimodal imaging features, including ultrasonography, color Doppler imaging, elastography, and contrast-enhanced MRI, thereby supplementing the limited available data on this rare entity.

## 1 Introduction

Testicular cancer is the most common solid malignancy in young men, accounting for approximately 1% of all cancers in men worldwide (1). Germ cell tumors constitute approximately 90%–95% of testicular malignancies, whereas sex cord-stromal tumors and other rare histological subtypes account for only a small proportion (2, 3). The incidence of testicular cancer varies substantially according to geographic region and ethnicity, with the highest rates reported in Northern and Western Europe, approximately 6.4 per 100,000, and lower rates in Africa and Asia, approximately 0.59 per 100,000 (1, 4).

Testicular intestinal-type adenocarcinoma is an exceedingly rare malignant neoplasm characterized by intestinal epithelial differentiation. Histologically, it typically demonstrates glandular, tubular, papillary, or trabecular architectures resembling colorectal adenocarcinoma and represents only a minute fraction of testicular malignancies (5). When intestinal-type adenocarcinoma morphology is encountered in the testis, careful distinction should be made among three diagnostic possibilities: somatic-type malignancy arising from a postpubertal germ cell tumor, for which chromosome 12p abnormalities provide important diagnostic evidence; primary testicular or paratesticular adenocarcinoma, which remains a diagnosis of exclusion; and metastatic adenocarcinoma, which must be excluded as a priority (6–8). These three entities differ substantially in their therapeutic strategies and prognostic implications (9–11).

A literature search was conducted in PubMed and Web of Science through July 2026. The search terms were “testicular intestinal-type adenocarcinoma” and “testicular adenocarcinoma with intestinal differentiation.” The inclusion criteria were as follows: 1) pathologically confirmed cases of testicular intestinal-type adenocarcinoma; 2) availability of complete clinical and/or imaging data; and 3) original articles published in English. To the best of our knowledge, only five English-language publications comprising six cases of testicular intestinal-type adenocarcinoma have been reported to date. Most previously reported tumors were relatively large, measuring >5 cm, which contrasts sharply with the subcentimeter, <1 cm, presentation in the present case. Moreover, none of these reports provided a detailed characterization of the imaging features of this tumor. Herein, we report a case of testicular intestinal-type adenocarcinoma without an identifiable extratesticular primary site in a 33-year-old man, with comprehensive multimodal imaging findings, and review the relevant literature.

## 2 Case presentation

A 33-year-old man was referred to our hospital on December 24, 2025, after a left testicular nodule was incidentally detected during a preconception health examination at an outside institution. The patient denied scrotal pain, swelling, fever, urinary symptoms, trauma, weight loss, changes in bowel habits, hematochezia, or other gastrointestinal complaints. He had no history of cryptorchidism, testicular trauma, infertility treatment, malignancy, or family history of colorectal cancer. His parents and grandparents had no history of gastrointestinal malignancy, inflammatory bowel disease, or polyposis syndrome.

Physical examination revealed a firm, non-tender, poorly mobile nodule approximately the size of a soybean in the medial upper-middle portion of the left testis. No abnormality was palpable in the right testis. Serum tumor markers were all within the reference ranges: β-human chorionic gonadotropin <1.20 mIU/mL, alpha-fetoprotein 2.65 ng/mL, and lactate dehydrogenase 124 U/L.

**Figure 1:**
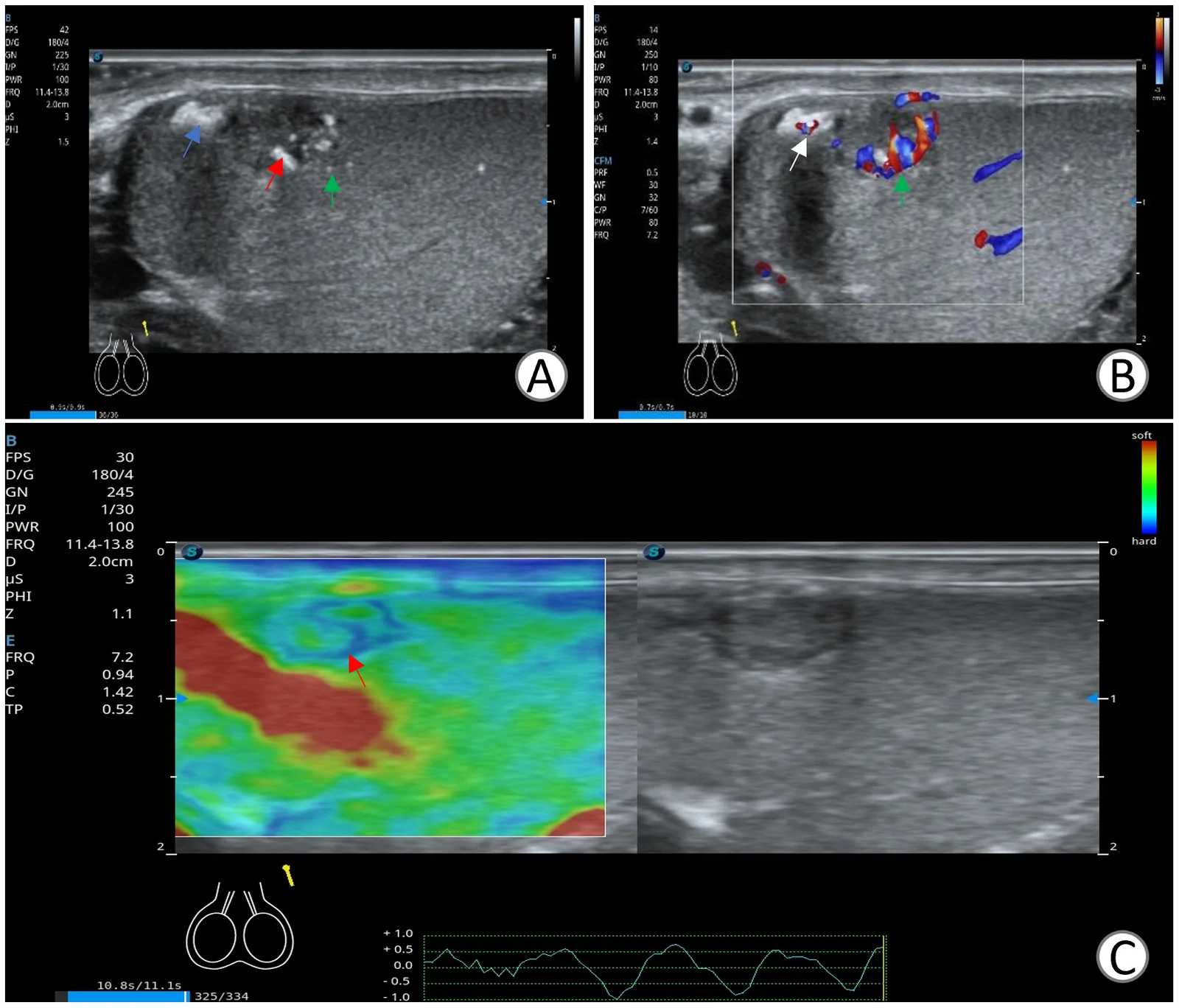
Ultrasonographic features of primary testicular intestinal-type adenocarcinoma. **(A)** Grayscale ultrasound shows an irregular, poorly marginated hypoechoic nodule in the upper-to-middle portion of the left testis. Peripheral echogenic foci, including punctate (green arrow), linear (red arrow), and clustered (blue arrow) types, are visible at the lesion margin, with posterior acoustic shadowing. **(B)** Color Doppler flow imaging demonstrates twinkle artifact posterior to the echogenic foci (white arrow), as well as abundant peripheral vascularity, with vessels extending into the lesion and forming a compact “ball-like” vascular configuration (green arrow). **(C)** Strain elastography shows increased stiffness at the lesion periphery.

Scrotal ultrasonography was performed using a SonoScape P60 ultrasound system with a transducer frequency of 11.3–13.8 MHz. The color Doppler flow imaging parameters were as follows: pulse repetition frequency, 0.5 kHz; velocity scale, 3 cm/s; and color gain, 32. Tissue stiffness was assessed using strain elastography. Ultrasonography demonstrated an irregular, ill-defined hypoechoic nodule in the upper-middle portion of the left testis, measuring approximately 9.7 × 6.8 × 6.3 mm. Multiple punctate, linear, and clustered hyperechoic foci were observed, predominantly at the periphery of the lesion, accompanied by distinct posterior acoustic shadowing and mild twinkling artifacts. Color Doppler flow imaging showed abundant peripheral vascular signals, with vessels extending into the lesion and forming a dense “ball-like” vascular configuration. Strain elastography demonstrated that the peripheral portion of the lesion was stiffer than the central portion (**Figure 1**; **Supplementary Videos S1, S2**; **Supplementary File S1**).

**Figure 2:**
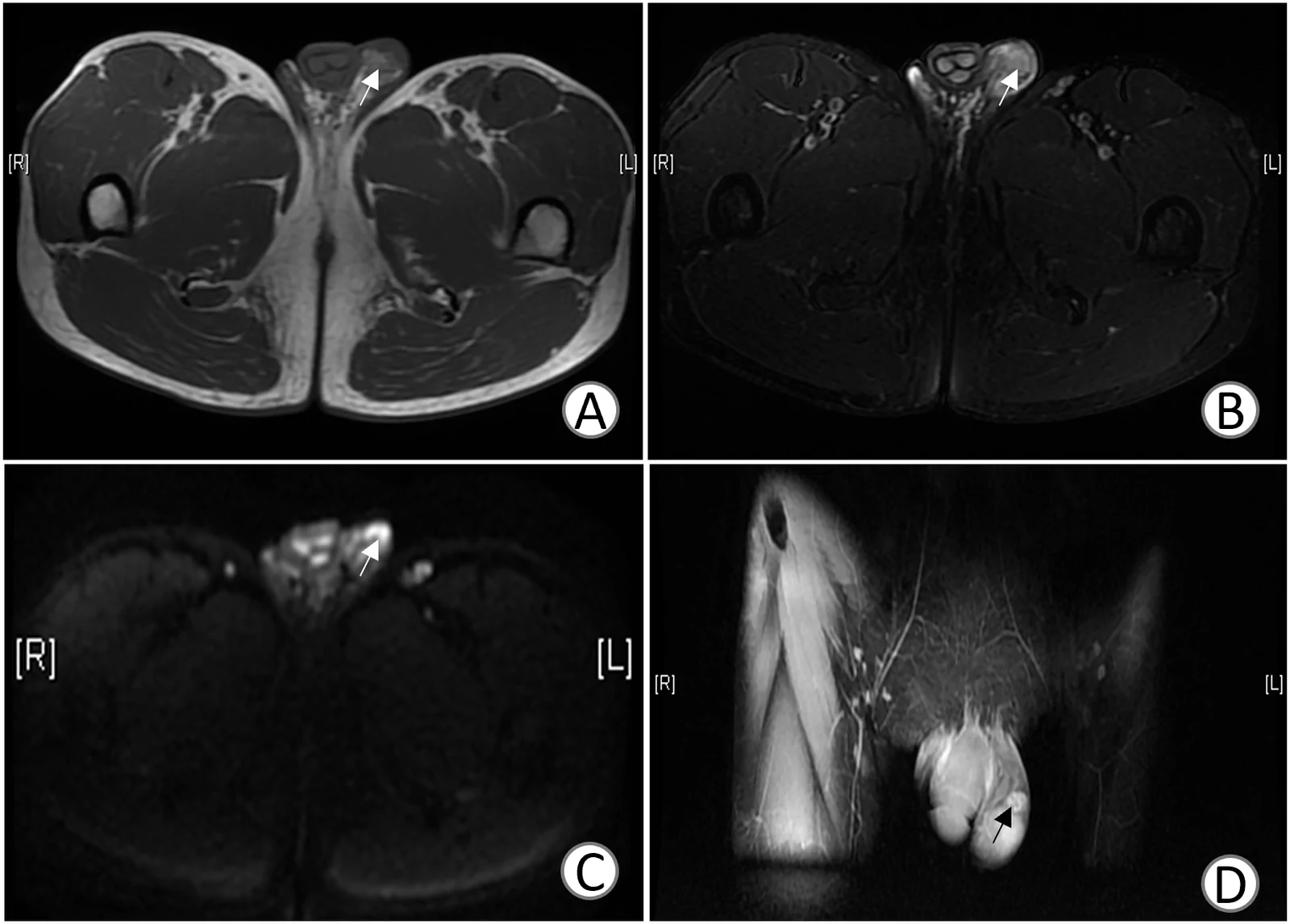
MRI features of primary testicular intestinal-type adenocarcinoma. **(A)** Axial T1-weighted image shows a hypointense nodule in the upper portion of the left testis. **(B)** Axial T2-weighted image shows corresponding hypointensity. **(C)** Axial diffusion-weighted image shows high signal intensity, consistent with restricted diffusion **(D)** Coronal fat-suppressed contrast-enhanced T1-weighted image shows marked peripheral-dominant enhancement.

Pelvic MRI was performed using a GE 1.5-T superconducting MRI scanner with an 8-channel phased-array body coil for signal acquisition. To reduce motion artifacts, the penis was positioned upward and fixed to the abdominal wall, and the scrotum was supported and immobilized with towels. The MRI protocol included axial T1-weighted fast spin-echo imaging, with repetition time of 400–500 ms, echo time of 12–20 ms, slice thickness of 3 mm, matrix of 256 × 256, and field of view of 20 cm; axial, coronal, and sagittal T2-weighted fast spin-echo imaging with frequency-selective fat suppression, with repetition time of 3000–5500 ms, echo time of 100–130 ms, slice thickness of 3 mm, matrix of 320 × 256, and field of view of 20 cm; axial diffusion-weighted imaging, with repetition time of 3800–4200 ms, echo time of 110–120 ms, slice thickness of 4 mm, and b values of 0 and 700 s/mm²; and axial dynamic contrast-enhanced MRI supplemented by coronal imaging, using a three-dimensional spoiled gradient-echo sequence, with repetition time of 4.7–9.0 ms, echo time of 2.3–4.1 ms, slice thickness of 3 mm, and flip angle of 35°.For contrast-enhanced imaging, gadolinium-based contrast agent Gd-DTPA was administered as an intravenous bolus via an antecubital vein at a dose of 0.1 mmol/kg and an injection rate of 2.5–3.0 mL/s, followed by a 20-mL saline flush. All dynamic contrast-enhanced MRI images were processed using automatic subtraction. RI confirmed a small nodule in the upper portion of the left testis, measuring approximately 8.6 × 5.3 mm. The lesion showed low signal intensity on both T1-weighted and T2-weighted images. Diffusion-weighted imaging demonstrated high signal intensity, with a corresponding apparent diffusion coefficient value of approximately 0.75×10−3 mm2/s0.75×10−3 mm2/s, consistent with restricted diffusion. After contrast administration, the lesion showed marked enhancement, predominantly along the periphery. No pelvic or retroperitoneal lymphadenopathy was identified (**Figure 2**).

Given the intratesticular location, hypoechoic appearance, vascularity, and restricted diffusion, a malignant testicular tumor was clinically suspected. Based on the patient’s age and imaging findings, seminoma was considered the leading preoperative diagnosis. However, the subcentimeter size, mixed peripheral calcifications, peripheral-predominant vascularity, increased peripheral stiffness, and peripheral rim enhancement were not typical features of seminoma. Leydig cell tumor, mixed germ cell tumor, and granulomatous inflammation were also considered in the preoperative differential diagnosis.

Before surgery, the patient was explicitly informed of the diagnostic uncertainty and malignant potential of the lesion. He was also informed that radical orchiectomy would be the standard surgical procedure if intraoperative frozen-section examination suggested malignancy. However, after fully understanding the potential risks, the patient and his wife firmly refused radical orchiectomy and insisted on partial resection only. After detailed counseling and written informed consent, partial orchiectomy was performed in accordance with the patient’s preference.

A 4-cm incision was made at the junction of the left inguinal region and scrotal root. The scrotum was accessed through the inguinal canal approach, and the spermatic cord was mobilized to the internal ring. The spermatic artery was not separately clamped. Intraoperatively, a well-encapsulated mass was identified at the upper pole of the left testis, without obvious adhesion to surrounding tissues. The mass was excised together with a small rim of adjacent normal testicular tissue and submitted for intraoperative frozen-section examination, which supported a malignant lesion. Because the patient and his family continued to firmly decline radical surgery, meticulous hemostasis was performed, a rubber drainage strip was placed, and the incision was closed layer by layer.

**Figure 3:**
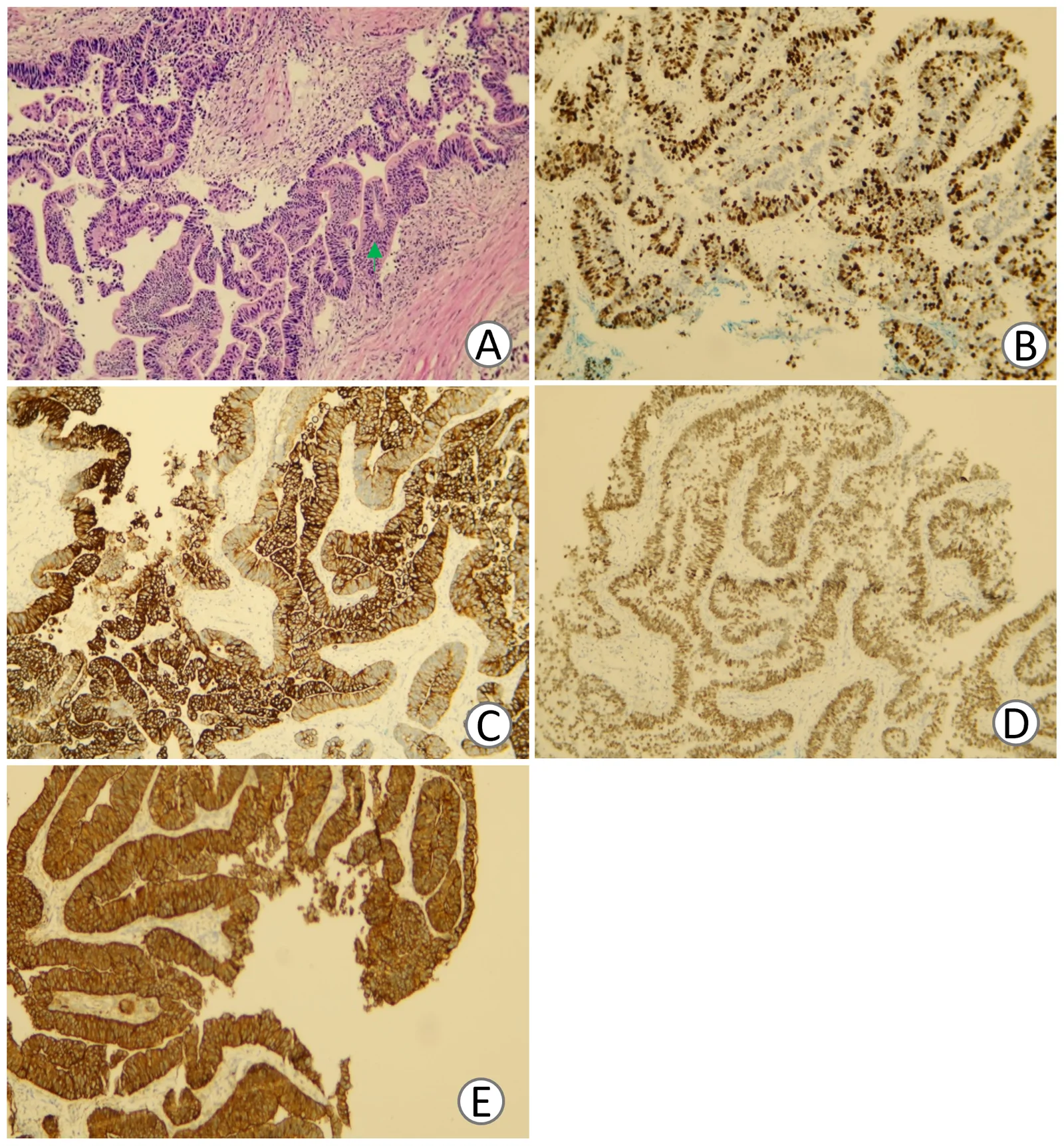
Histopathological and immunohistochemical features of the testicular intestinal-type adenocarcinoma. **(A)** Hematoxylin and eosin (H&E) staining shows papillary and glandular-papillary structures lined by columnar epithelium (green arrow). Original magnification: 200×. **(B)** Immunohistochemistry shows a Ki-67 proliferation index of approximately 60%. Original magnification: 100×. **(C)** The tumor cells are positive for CK20. Original magnification: 100×. **(D)** The tumor cells are positive for SATB2. Original magnification: 100×. **(E)** The tumor cells are positive for Villin. Original magnification: 100×.

Grossly, the specimen consisted of a gray-yellow to gray-white tissue fragment measuring approximately 10 × 10 × 9 mm. Microscopically, the tumor demonstrated papillary and glandular-papillary structures lined by columnar epithelium. The papillae varied in size and shape and focally coalesced into complex glandular structures (**Figure 3A**). The tumor cell nuclei were round to oval. Mitotic figures were observed, averaging approximately 5–8 per high-power field. No obvious necrosis was identified, and focal mild mucin secretion was present. Lymphovascular invasion and perineural invasion were absent. The surgical margin was negative, with the tumor located approximately 2.5 mm from the nearest margin. The tumor was confined to the testicular parenchyma, without involvement of the rete testis and without invasion through the tunica albuginea or tunica vaginalis. No teratomatous component, yolk sac tumor differentiation, or germ cell neoplasia *in situ* was identified microscopically.

Immunohistochemical staining showed that the tumor cells were positive for CK, CK20, CD10, Villin, and SATB2, with focal positivity for CK7. The tumor cells were negative for CK5/6, Calretinin, WT-1, PSA, PSAP, OCT3/4, SALL4, and PAX8. The Ki-67 labeling index was approximately 60%. p53 immunohistochemistry, performed using the DO-7 clone, demonstrated diffuse strong nuclear positivity in approximately 90%–95% of tumor cells, whereas the surrounding non-neoplastic cells showed scattered weak-to-moderate nuclear staining. Overall, the morphological and immunophenotypic features supported a diagnosis of adenocarcinoma with intestinal differentiation (**Figures 3B–E**).

**Figure 4:**
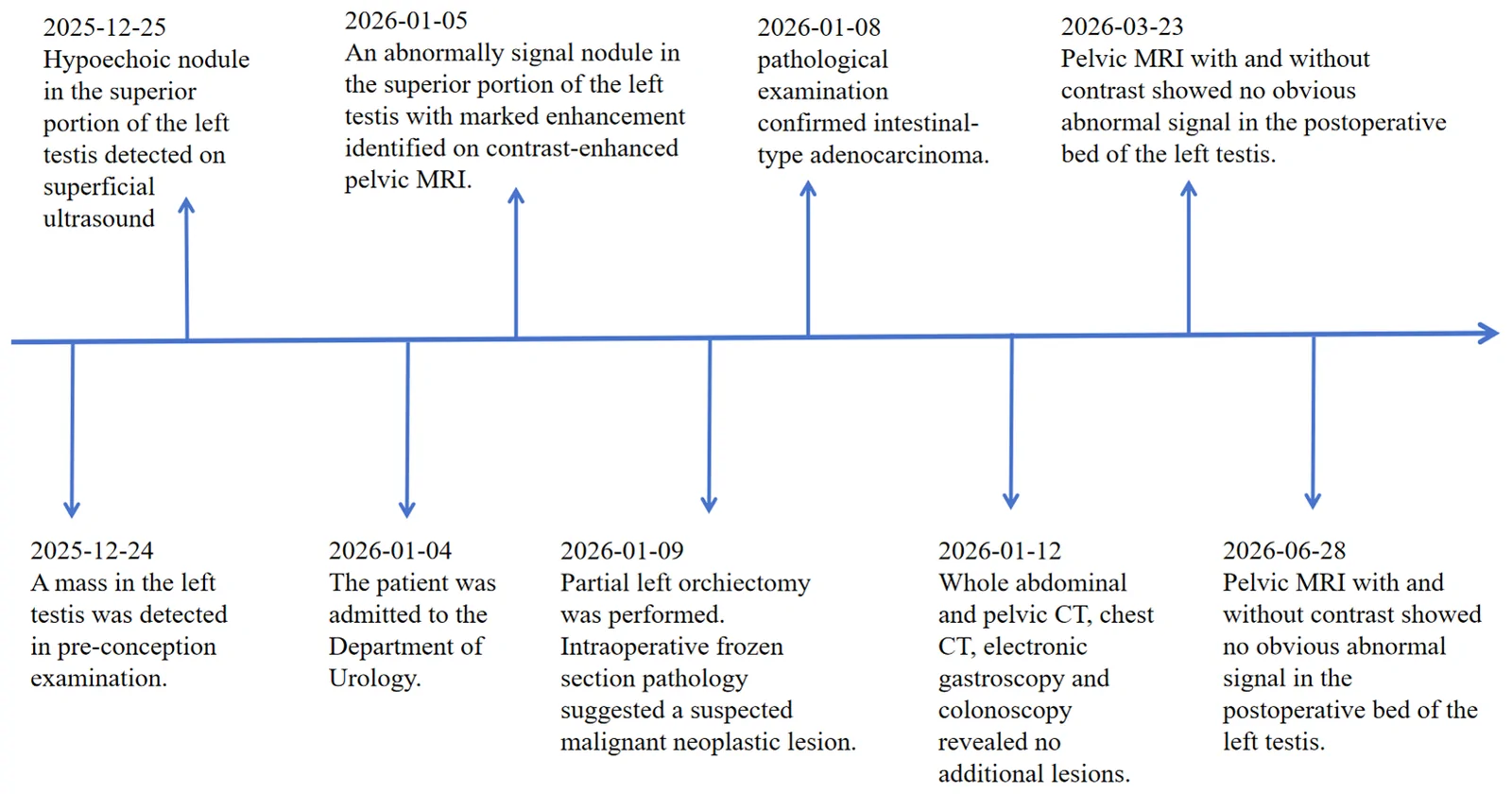
Complete clinical timeline of the patient.

To evaluate whether the testicular intestinal-type adenocarcinoma originated from another site, the patient subsequently underwent non-contrast and contrast-enhanced whole-abdominal CT covering the abdomen, pelvis, and retroperitoneum, as well as chest CT, upper gastrointestinal endoscopy, and colonoscopy. No abnormalities were detected. The final clinical diagnosis was testicular intestinal-type adenocarcinoma without an identifiable extratesticular primary site. However, because the lesion was removed by partial rather than radical orchiectomy, complete pathological staging, including definitive pT classification, could not be reliably established. Therefore, no formal staging system was applied for final stage classification. The patient underwent regular postoperative MRI surveillance, which showed no evidence of recurrence or extratesticular disease. The complete clinical timeline is shown in **Figure 4**.

## 3 Discussion

This case illustrates a rare but clinically important diagnostic challenge: testicular intestinal-type adenocarcinoma may present as a small intratesticular nodule and show imaging features that overlap with those of seminoma. The patient was a 33-year-old man with an incidentally detected lesion, normal serum testicular tumor markers, and a tumor measuring less than 1 cm. If imaging findings are interpreted in isolation, these features may lead to underestimation of the malignant potential. Therefore, multimodal imaging–pathology correlation is essential for recognizing this rare entity.

Testicular cancer is the most common malignancy among men aged 14–44 years (1). The most common clinical manifestation is a painless testicular mass or painless testicular enlargement, although approximately 10% of patients may be asymptomatic. Some patients may present with scrotal heaviness or dull pain, and a minority may experience acute pain secondary to intratumoral hemorrhage (12).

Regarding tumor markers, approximately 60% of patients with non-seminomatous germ cell tumors show elevated AFP and/or β-hCG levels. In pure seminoma, AFP is usually normal, whereas β-hCG may be mildly elevated. LDH has low sensitivity and lacks specificity; its level is generally associated with tumor burden, and it may remain completely normal in patients with early-stage or localized disease (13).

In the present case, the patient was a 33-year-old man who presented with an incidentally detected painless small testicular nodule. Ultrasonography and MRI suggested a solid intratesticular lesion, whereas serum AFP, β-hCG, and LDH levels were all within the normal ranges. These findings are consistent with the general clinical features of testicular cancer and are also in line with previously reported cases.

Histologically, testicular intestinal-type adenocarcinoma is typically characterized by papillary and glandular-papillary structures lined by columnar epithelium (5). The tumor cell nuclei are usually round to oval, and mitotic figures may be observed, as in the present case, with an average of 5–8 mitoses per high-power field (6). Immunohistochemistry is crucial for diagnosis. Positivity for CK20, Villin, and SATB2 supports intestinal differentiation in intestinal-type adenocarcinoma (14). OCT3/4 and SALL4 are typically positive in seminoma and embryonal carcinoma but are usually negative in somatic-type malignant transformation (15). In the present case, the histopathological and immunohistochemical profile, namely CK20+/Villin+/SATB2+ and OCT3/4−/SALL4−, was consistent with intestinal-type adenocarcinoma.

Among the reported cases summarized in **Table 1**, testicular intestinal-type adenocarcinoma predominantly involved the left testis, with left-sided disease reported in 5 of 6 cases; the only right-sided case was reported in Cureus. The tumor often presented as a mass larger than 5 cm in diameter in previous reports, including 56 mm in case 1 and 70 mm in case 2 reported by Rozsvai et al. in 2025, and a recurrent lesion measuring 134 mm reported by Oosterhuis et al. in 2013 (6, 16–19). In contrast, the present tumor arose in the left testis and measured less than 1 cm, representing an extremely rare subcentimeter presentation.

**Table 1:** Summary of previously reported key cases of testicular intestinal-type.

Author/year	Age	Histology	Other GCT components	12p status	Tumor size	Imaging	Surgery	Stage	Treatment	Follow-up
Rozsvai et al., 2025 (6) (Case 1)	63	Intestinal-type adenocarcinoma (somatic-type malignancy)	N/A	12p gain (FISH+)	56 mm (primary)	US: suspicious	Left radical orchiectomy	pT2 (lung + retroperitoneal metastases)	CAPOX + bevacizumab	Stable disease at 24 months
Rozsvai et al., 2025 (6) (Case 2)	52	Intestinal-type adenocarcinoma (somatic-type malignancy)	N/A (original GCT diagnosed at 35, removed 2007)	12p gain (FISH+)	70 mm (retroperitoneal metastasis)	N/A	Left radical orchiectomy (primary, 2007) + RPLND (recurrence)	Late recurrence (16 years)	CAPOX + bevacizumab	Stable disease ≥9 months
Oosterhuis et al., 2013 (17)	47 (primary at 28)	Intestinal-type adenocarcinoma + low-grade leiomyosarcoma	N/A (non-seminoma, 19 years earlier)	N/A	~134×119×60 mm (retroperitoneal recurrence)	N/A	Left radical orchiectomy (primary)	Late recurrence (19 years)	N/A	Alive with disease at 21 years
Cureus, 2022	44	Adenocarcinoma (somatic-type malignancy)	N/A	N/A	13 mm (retroperitoneal LN)	CT/PET: solitary retroperitoneal LN, SUVmax 3	Right radical orchiectomy + RPLND + metastasis resection	IIA → late recurrence (10 years)	EP ×1 cycle (not completed)	Post-recurrence resection
Yu et al., 2023 (18)	55	Intestinal-type adenocarcinoma (mucinous/signet ring)	N/A (metastatic, cecum primary)	N/A	N/A	US: no flow (misdiagnosed as torsion)	Left orchiectomy (misdiagnosed)	IV	Declined chemo	Survived 18 months
Koseoglu et al., 2007 (19)	N/A	Adenocarcinoma (from mature teratoma)	N/A	N/A	N/A	N/A	Orchiectomy (side N/A)	N/A	N/A	N/A
Present case	33	Intestinal-type adenocarcinoma	N/A (GCNIS– by morphology)	N/A (not tested, core limitation)	<1 cm (US 9.7mm, MRI 8.6mm)	Multimodality imaging (US+CDFI+elastography+MRI)	Left partial orchiectomy	Localized (imaging N0M0, no formal stage)	No adjuvant therapy	6 months (ongoing)

Only a few previous cases have described the ultrasonographic features of this tumor, mainly as a hypoechoic mass (6), occasionally with calcification (6, 19). In the metastatic intestinal-type adenocarcinoma reported by Yu et al. in 2023, ultrasonography showed no detectable blood flow signal (18). However, previous reports did not describe elastographic findings or contrast-enhanced MRI features (6, 16–19). To our knowledge, the present case is the first to systematically document the multimodal imaging characteristics of testicular intestinal-type adenocarcinoma. In this case, ultrasonography demonstrated a hypoechoic nodule in the left testis with multiple marginal hyperechoic foci, abundant peripheral vascularity, and increased peripheral stiffness on elastography. MRI showed low signal intensity on both T1-weighted and T2-weighted images, high signal intensity on diffusion-weighted imaging, restricted diffusion, and marked peripheral enhancement after contrast administration. Pathologically, these imaging findings may be attributable to the glandular-papillary architecture of the tumor, which may lead to gradual accumulation of contrast agent in the peripheral tumor region, resulting in persistent peripheral-dominant enhancement.

Testicular intestinal-type adenocarcinoma is exceedingly rare and should be differentiated from seminoma, mixed germ cell tumor, sex cord-stromal tumor, and metastatic adenocarcinoma.

### Seminoma

Seminoma is the most common malignant testicular tumor. On ultrasonography, it typically appears as a relatively heterogeneous hypoechoic mass with abundant internal vascularity, and MRI may demonstrate restricted diffusion. However, seminomas are usually larger, with a reported mean diameter of approximately 38 mm (20). Calcifications, when present, are generally punctate or psammomatous microcalcifications (21), and serum β-hCG may be elevated. In the present case, the lesion was subcentimeter in size and showed mixed peripheral calcifications, which are not typical features of seminoma.

### Mixed germ cell tumor

Mixed germ cell tumors are usually larger, with a reported maximum diameter of approximately 50.77 ± 23.31 mm. On ultrasonography, they often present as cystic-solid mixed echogenic or hypoechoic masses with ill-defined margins and abundant vascularity in the solid components (22). On MRI, they typically show heterogeneous high signal intensity on T2-weighted imaging, with internal necrosis, hemorrhage, or cystic degeneration. Diffusion-weighted imaging shows restricted diffusion with a markedly decreased ADC value (23). Serum AFP and/or β-hCG levels are frequently elevated. In the present case, the lesion was small, serum tumor markers were normal, and no typical necrosis, hemorrhage, or cystic change was observed.

### Sex cord-stromal tumor

Sex cord-stromal tumors include Leydig cell tumors. On ultrasonography, Leydig cell tumors typically present as well-defined small hypoechoic nodules, with a reported median size of 7.0 mm. Color Doppler imaging shows hypervascularity in approximately 95% of lesions, often characterized by an encircling vascular pattern or mixed central and peripheral vascularity. On MRI, Leydig cell tumors usually show markedly low signal intensity on T2-weighted imaging, followed by intense early homogeneous enhancement and rapid or delayed washout kinetics (24). In contrast, the present lesion was irregular and ill-defined, with mixed peripheral calcifications, which is inconsistent with the typical features of Leydig cell tumor. Immunohistochemically, Leydig cell tumors are usually positive for Calretinin, whereas the present tumor was negative, further arguing against this diagnosis.

### Metastatic adenocarcinoma

Metastatic adenocarcinoma must be excluded whenever intestinal differentiation is identified in a testicular tumor. Testicular metastases are extremely rare and occur more commonly in older patients, with a reported median age of approximately 51 years. On imaging, metastatic lesions often appear as homogeneous hypoechoic masses on ultrasonography (25). On MRI, they may show peripheral rim enhancement with central necrosis, which typically lacks enhancement and diffusion restriction (26). In the present case, the lesion was solitary, the patient was young, 33 years old, and imaging demonstrated a solid peripherally enhancing lesion with calcification rather than a necrotic ring-enhancing mass. Moreover, systemic evaluation was negative. These findings do not support the diagnosis of metastatic adenocarcinoma.

Radical inguinal orchiectomy remains the standard treatment for malignant testicular tumors. Traditionally, testis-sparing surgery, or partial orchiectomy, has been reserved for patients with bilateral tumors, a solitary testis, or severely impaired contralateral testicular function (1–3, 27). However, the management of small testicular masses, defined as lesions measuring ≤20 mm, has evolved in recent years. Studies have shown that up to 86% of small testicular masses are benign, indicating that radical orchiectomy may constitute overtreatment in selected patients (28). Both the 2026 EAU Guidelines and the 2024 Delphi consensus on the management of small testicular masses indicate that testis-sparing surgery may be considered in patients with small testicular masses, with individualized decision-making based on testicular cancer risk factors, fertility status, contralateral testicular status, and ultrasonographic characteristics (27, 28).

In the present case, the lesion was a serum marker-negative subcentimeter nodule, <1 cm, meeting the definition of a small testicular mass. The patient and his spouse had not yet had children and strongly wished to preserve testicular tissue. The patient was explicitly informed before surgery that radical orchiectomy would be the standard procedure if intraoperative frozen-section examination suggested malignancy. Although frozen-section examination supported a malignant lesion, the patient and his spouse continued to firmly decline radical orchiectomy after fully understanding the potential oncological risks. Therefore, partial orchiectomy was performed after written informed consent was obtained. The patient was advised to undergo close postoperative surveillance and was informed that radical orchiectomy would still be required in the event of recurrence. This decision reflected a careful balance between oncological safety and the patient’s desire for functional preservation and represents an individualized, patient-centered clinical approach.

The responsiveness of testicular intestinal-type adenocarcinoma to radiotherapy and chemotherapy remains unclear, and current evidence is insufficient to demonstrate that adjuvant therapy improves prognosis (1, 12). In the present case, no extratesticular disease was detected, corresponding to an imaging-based N0M0 status. The patient did not receive adjuvant therapy, and no recurrence was observed during 6 months of follow-up.

The main strengths of this report are as follows. First, to our knowledge, this is the first systematic description of the multimodal imaging features of testicular intestinal-type adenocarcinoma, including ultrasonography, color Doppler imaging, elastography, and MRI, thereby addressing a gap in the existing literature. Second, combined ultrasonographic and MRI assessment revealed a peripheral rim enhancement pattern, which may provide a useful clue for preoperative imaging-based differential diagnosis. Third, this case demonstrates the complete diagnostic pathway from imaging suspicion and intraoperative frozen-section examination to pathological confirmation and systematic exclusion of metastatic adenocarcinoma, emphasizing the clinical principle that an extratesticular primary site must be excluded when intestinal differentiation is identified in a testicular tumor.

This report also has several limitations. First, as a single-case report, it lacks support from a large sample size, and the findings cannot be generalized to all patients with testicular intestinal-type adenocarcinoma. Second, 12p FISH or next-generation sequencing was not performed; therefore, a germ cell origin could not be molecularly confirmed. This represents the most important diagnostic limitation. Third, CDX2 immunohistochemistry could not be performed because of insufficient tissue, and complete pathological staging could not be established using the partial orchiectomy specimen. Fourth, the follow-up period was only 6 months, which is insufficient to evaluate long-term prognosis and the risk of local recurrence. Continued long-term surveillance is therefore necessary.

## 4 Conclusion

In conclusion, we report a case of testicular intestinal-type adenocarcinoma without an identifiable extratesticular primary site in a 33-year-old man and describe its clinical, multimodal imaging, and pathological features, thereby supplementing the limited literature on this rare entity. Based on the few previously published cases, the biological behavior and optimal management of testicular intestinal-type adenocarcinoma remain uncertain. Because its clinical and imaging features are nonspecific, misdiagnosis may occur. Detailed case reports are therefore valuable for improving clinical recognition and differential diagnosis.

## Supplementary material

Supplementary Video 1 Dynamic grayscale ultrasound of the left testicular nodule, showing a hypoechoic lesion with mixed peripheral calcifications.

Supplementary Video 2 Dynamic color Doppler ultrasound of the left testicular nodule, demonstrating abundant peripheral vascularity with a “ball-like” configuration.

Supplementary File S1 Completed CARE checklist.
