# Supplementary material for: Testicular intestinal-type adenocarcinoma without an identifiable extratesticular primary site: a case report and literature review
Source: Front Oncol. 2026 Sep 3;16:1926619. doi: 10.3389/fonc.2026.1926619 (PMC13581793; doi:10.3389/fonc.2026.1926619)
Supplement: Supplementary file 1 [file Supplementaryfile1.docx]

**CARE Checklist of information to include when writing a case report**

| Topic | Item | Checklist item description | Reported in Section |
| --- | --- | --- | --- |
| **Title** | 1 | The diagnosis or intervention of primary focus followed by the words "case report" | Title |
| **Key Words** | 2 | 2 to 5 key words that identify diagnoses or interventions in this case report, including "case report" | Keywords |
| **Abstract** | 3a | Introduction: What is unique about this case and what does it add to the scientific literature? | Abstract (Background) |
|  | 3b | Main symptoms and/or important clinical findings | Abstract (Case presentation) |
|  | 3c | The main diagnoses, therapeutic interventions, and outcomes | Abstract (Case presentation) |
|  | 3d | Conclusion—What is the main "take-away" lesson(s) from this case? | Abstract (Conclusion) |
| **Introduction** | 4 | One or two paragraphs summarizing why this case is unique (may include references) | Introduction |
| **Patient Information** | 5a | De-identified patient specific information | Case Presentation |
|  | 5b | Primary concerns and symptoms of the patient | Case Presentation |
|  | 5c | Medical, family, and psycho-social history including relevant genetic information | Case Presentation |
|  | 5d | Relevant past interventions with outcomes | Case Presentation |
| **Clinical Findings** | 6 | Describe significant physical examination (PE) and important clinical findings | Case Presentation |
| **Timeline** | 7 | Historical and current information from this episode of care organized as a timeline | Timeline (Figure 4) |
| **Diagnostic Assessment** | 8a | Diagnostic testing (such as PE, laboratory testing, imaging, surveys) | Diagnostic Assessment |
|  | 8b | Diagnostic challenges (such as access to testing, financial, or cultural) | Not applicable (N/A) |
|  | 8c | Diagnosis (including other diagnoses considered) | Diagnostic Assessment |
|  | 8d | Prognosis (such as staging in oncology) where applicable | Not formally staged (partial orchiectomy) |
| **Therapeutic Intervention** | 9a | Types of therapeutic intervention (such as pharmacologic, surgical, preventive, self-care) | Therapeutic Intervention |
|  | 9b | Administration of therapeutic intervention (such as dosage, strength, duration) | Therapeutic Intervention – partial orchiectomy |
|  | 9c | Changes in therapeutic intervention (with rationale) | Not applicable (N/A) |
| **Follow-up and Outcomes** | 10a | Clinician and patient-assessed outcomes (if available) | Follow-up and Outcomes |
|  | 10b | Important follow-up diagnostic and other test results | Follow-up and Outcomes |
|  | 10c | Intervention adherence and tolerability (How was this assessed?) | Follow-up and Outcomes – patient declined adjuvant therapy |
|  | 10d | Adverse and unanticipated events | None occurred |
| **Discussion** | 11a | A scientific discussion of the strengths AND limitations associated with this case report | Discussion |
|  | 11b | Discussion of the relevant medical literature with references | Discussion |
|  | 11c | The scientific rationale for any conclusions (including assessment of possible causes) | Discussion |
|  | 11d | The primary "take-away" lessons of this case report (without references) in a one paragraph conclusion | Discussion / Conclusion |
| **Patient Perspective** | 12 | The patient should share their perspective in one to two paragraphs on the treatment(s) they received | Patient Perspective |
| **Informed Consent** | 13 | Did the patient give informed consent? Please provide if requested | Ethics Statement – yes, written informed consent obtained |
